# Association Between Renal Dysfunction and Low HDL Cholesterol Among the Elderly in China

**DOI:** 10.3389/fcvm.2021.644208

**Published:** 2021-05-12

**Authors:** Aijun You, Yaxin Li, Brian Tomlinson, Longfei Yue, Kaijie Zhao, Huimin Fan, Zhongmin Liu, Yuzhen Zhang, Liang Zheng

**Affiliations:** ^1^Shanghai Heart Failure Research Center, Shanghai East Hospital, Tongji University School of Medicine, Shanghai, China; ^2^Research Center for Translational Medicine, Shanghai East Hospital, Tongji University School of Medicine, Shanghai, China; ^3^Department of Epidemiology and Public Health, Tongji University School of Medicine, Shanghai, China; ^4^Faculty of Medicine, Macau University of Science and Technology, Macau, China; ^5^Anshun People's Hospital, Guizhou, China; ^6^Community Health Service Center, Shanghai, China

**Keywords:** renal dysfunction, dyslipidemia, estimated glomerular filtration rate, high-density lipoprotein cholesterol, cardiovascular prevention

## Abstract

**Objective:** Chronic kidney disease (CKD) and cardiovascular disease (CVD) have a high morbidity and mortality among the elderly. Low levels of high-density lipoprotein cholesterol (HDL-C), a traditional risk marker for CVD, are common in CKD patients. Little is known about the association of low HDL-C with renal dysfunction in the community dwelling population.

**Methods:** This was a population-based cross-sectional study included 4,753 participants enrolled in a prospective study, the Shanghai Elderly Cardiovascular Health (SHECH) study. Estimated glomerular filtration rate (eGFR), calculated by the Chinese Modification of Diet in Renal Disease (C-MDRD equation), was used to assess renal dysfunction. Associations between renal dysfunction and low HDL-C were evaluated using multiple logistic regression models and restricted cubic splines.

**Results:** Of 4,649 individuals who met inclusion criteria, 620 (13.34%) had low HDL-C at <40 mg/dl. In the fully adjusted model, lower eGFR of <60 ml/min/1.73 m^2^ (OR, 2.03; 95% CI, 1.21–3.43) and marginal eGFR of 60 to 90 ml/min/1.73 m^2^ (OR, 1.26; 95% CI, 1.01–1.58) were significantly associated with low HDL-C, compared with normal eGFR of ≥90 ml/min/1.73 m^2^. Moreover, consistent findings were obtained in subsidiary analyses using the Chronic Kidney Disease Epidemiology Collaboration (CKD-EPI) equation. Fully adjusted cubic spline models indicated a significant dose-response relationship between eGFR and low HDL-C (*P* for non-linearity, 0.356).

**Conclusion:** In this general elderly population, renal dysfunction was independently and significantly associated with low HDL-C, and the prevalence of low HDL-C increased with decreasing eGFR, such that even slight changes in renal function may be associated with altered lipid levels.

## Introduction

China has gained remarkable achievements in controlling the burden of cardiovascular disease (CVD) over the past two decades, and the standardized mortality rate has shown a significant decrease (1). However, the disease burden due to CVD remains severe in patients with chronic kidney disease (CKD) (2). The China Kidney Disease Network (CK-NET) annual report shows that the prevalence of CVD in CKD patients in China is as high as 50.8% (3). CVD is the leading cause of death in CKD patients at different stages of CKD (4), especially in patients with end-stage renal disease (ESRD), the CVD mortality rate is 10–20 times higher than that of the general population (5). On the other hand, population aging is increasing with socioeconomic development. It is estimated that by 2025, there will be 300 million people aged 60 and above in China (6). Studies show that the disease burden in CKD patients increases with age (7, 8), and nearly one-half of CKD patients were aged 60 years or older (3). Therefore, it is imperative to prevent CVD events in the elderly population, especially those with CKD.

High-density lipoprotein cholesterol (HDL-C) is a traditional protective factor for CVD (9), which was thought to achieve its protective effect by mediating the reverse cholesterol transport pathway (10, 11), but it is known to decrease significantly in CKD patients (12, 13). The Framingham Heart Study showed that for every 1 mg/dl (0.026 mmol/L) increase in HDL-C, CVD risk was significantly reduced by 2–3% (14). Since then, more and more epidemiological studies have expanded and validated the findings (15). Previous studies have observed that HDL-C may be related to renal function in adults (16–19). However, the association of low HDL-C with renal dysfunction has not been well-defined, especially in elderly subjects living in the community in China. Since HDL-C changes might also be related to age, gender, body weight, physical activity, medication, disease, etc. (20), the independent association between HDL-C and renal dysfunction in the community elderly population should be estimated after adjusting for these potential confounders.

## Methods

### Study Population

**Figure 1:**
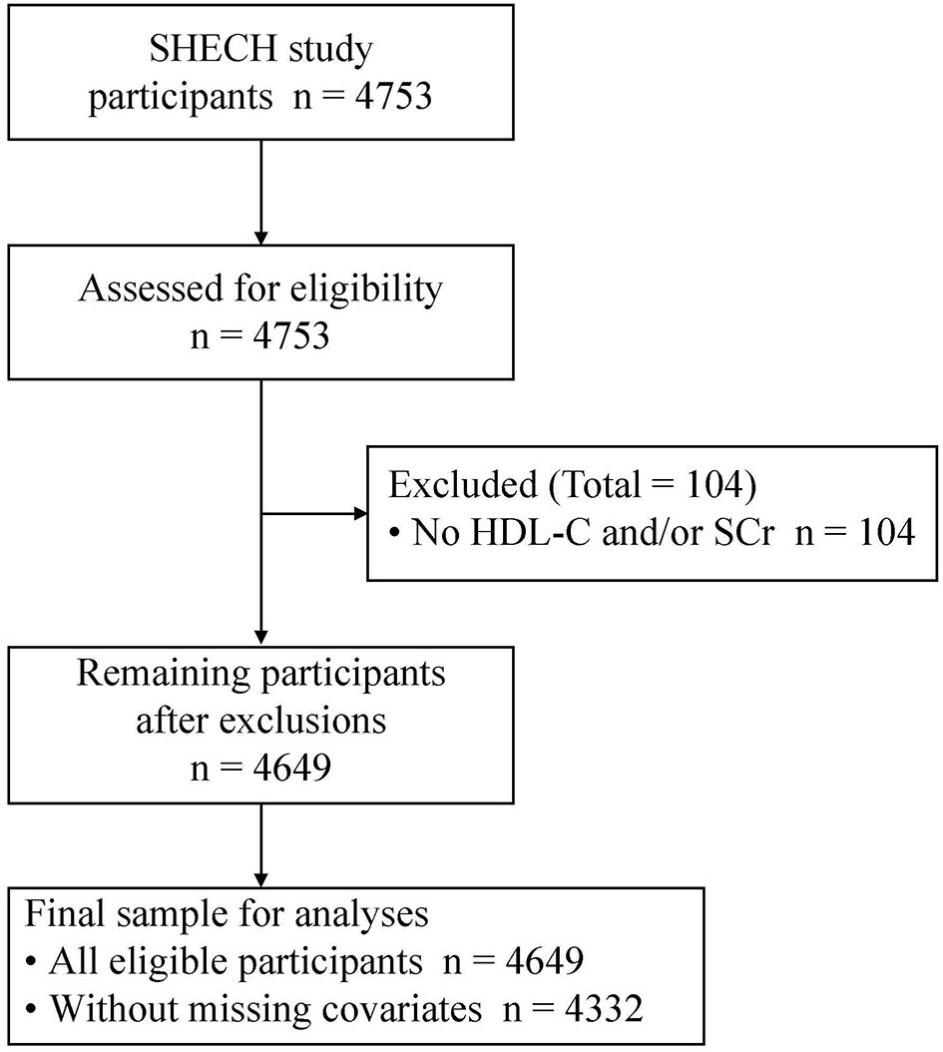
Study flow. HDL-C, high-density lipoprotein cholesterol; SCr, serum creatinine; SHECH, Shanghai Elderly Cardiovascular Health.

The Shanghai Elderly Cardiovascular Health (SHECH) study is a multistage cluster sampling survey of community non-institutionalized residents aged 60 years and older conducted since 2013. Its design details have been described previously (21). Briefly, 4,753 participants aged 60 to 104 years were recruited in the community during 2017 and underwent a comprehensive health screening at Shanghai East Hospital. Participants underwent blood tests after fasting for at least 10 h overnight, and blood samples were sent to the Blood Laboratory of Tongji Medical School affiliated Shanghai East Hospital for measurement within 2 h. In the current analysis, 104 participants were excluded due to missing both HDL-C and serum creatinine (SCr) measurements, so 4,649 participants were included (Figure 1).

### Data Collection

Participant information was collected through standardized questionnaires, including demographics, lifestyles, medication and disease history, etc. It was recoded if postmenopausal females were taking hormone replacement therapy as estrogen and/or progestin.

Body mass index (BMI) was calculated as weight in kilograms divided by height in meters squared. Abdominal obesity was defined as a waist circumference of >90 cm in males and >80 cm in females (22). Creatinine clearance (CCr) was determined by the Cockcroft-Gault equation (23). Current smoking was defined as smoking more than 100 cigarettes in a lifetime and still smoking. Current drinking as drinking 1 or more alcoholic beverages each week during the previous year. Physical activity was considered active if at least 4 days of exercise or recreational activities were performed per week and more than 30 min/day.

Hypertension was defined as an average of two measurements of systolic blood pressure of ≥140 mmHg, or diastolic blood pressure of ≥90 mmHg, or normal blood with concomitant use of antihypertensive agents (24). Diabetes was defined as fasting plasma glucose of ≥7.0 mmol/L or current use of insulin or oral antidiabetic agents. Atherosclerotic cardiovascular disease (ASCVD) was defined as a history of myocardial infarction, stable or unstable angina, coronary or other arterial revascularization, stroke, transient ischemic attack, or atherosclerotic peripheral artery disease (25). Liver dysfunction was defined as aspartate aminotransferase of >40 U/L, alanine aminotransferase of >50 U/L, or a history of liver disease. All disease histories were confirmed by reviewing the outpatient medical records of primary care in the community health centers.

Two seated blood pressure measurements using a mercury sphygmomanometer after at least 5 min of quiet rest were obtained by trained and certified staff who followed a standard protocol, with the average of two measurements used for the analysis. Hemoglobin AIc was measured using high-performance liquid chromatography (Tosoh Corporation, Tokyo, Japan). SCr, uric acid (UA), urea nitrogen (UN), lipids, and glucose were measured using a biochemical autoanalyzer (Cobas 8000, Roche Diagnostics, Mannheim, Germany).

### Outcome Ascertainment

In this study, estimated glomerular filtration rate (eGFR) was used to assess renal dysfunction. eGFR was calculated using the Chinese Modification of Diet in Renal Disease (C-MDRD) equation where eGFR (ml/min/1.73 m^2^) = 186 * SCr (mg/dl)^−1.154^ * age (years)^−0.203^ * 0.742 (if female) * 1.233 (if Chinese) (26) [with subsidiary analyses using eGFR calculated with the Chronic Kidney Disease Epidemiology Collaboration (CKD-EPI) equation (27)].

### Statistical Analyses

The study outcome was low HDL-C, which was defined as <40 mg/dl according to Chinese guidelines (28). Baseline characteristics of participants were summarized as mean [standard deviation (SD)] or median [interquartile range (IQR)] for continuous variables, and frequency (percentage) for categorical variables.

Statistical significance was tested using Student's *t* test, Welch's *t* test, and Mann–Whitney *U* test for normally or skewed distributed continuous variables and Chi-square test for categorical variables. Pearson's correlation coefficients were calculated to initially assess the correlation between HDL-C and renal function.

Multiple logistic regression models were performed to evaluate the relationship between eGFR and low HDL-C. Three models were fitted: Model I: unadjusted; Model II: adjusted for age, gender, and BMI; and Model III: as for Model II plus smoking, drinking, physical activity, β-blocker, statin, diabetes, liver dysfunction, ASCVD and triglycerides. Restricted cubic splines with three knots at the 25th, 50th, and 75th percentiles were used to simulate the association of eGFR with low HDL-C.

All *P*-values were two sided, and statistical significance was defined as *P* < 0.05. Analyses were performed using SAS, version 9.4 (SAS Institute, Inc., Cary, NC, USA).

## Results

### Baseline Characteristics

Among 4,649 participants, 2,090 (44.96%) were males, 1,640 (35.28 %) had diabetes, and 224 (4.82%) had liver dysfunction. The mean (SD) age was 72 (6) years; the mean (SD) eGFR was 101.77 (22.56) and 77.27 (14.35) ml/min/1.73 m^2^ for the C-MDRD and CKD-EPI equations, respectively; the mean (SD) HDL-C was 55 (16) mg/dl. Low HDL-C was observed in 620 participants (13.34%). Compared with those with non-low HDL-C, participants with low HDL-C were more likely to be males, obese, current smokers, hypertensive, diabetic, and ASCVD patients (Table 1).

**Table 1:** Baseline characteristics of participants according to low HDL-C status.

**Characteristics**	**All (*n* = 4,649)**	**Low HDL-C**		***P-*value**
		**Yes (*n* = 620)**	**No (*n* = 4,029)**	
Age (years)	72 (6)	72 (6)	72 (6)	0.560
Male	2,090 (44.96)	371 (59.84)	1,719 (42.67)	<0.001
BMI (kg/m^2^)	24.68 (3.46)	25.68 (3.03)	24.52 (3.50)	<0.001
WC (cm)	87.84 (12.22)	90.94 (10.71)	87.37 (12.37)	<0.001
Abdominal obesity	2,527 (54.36)	369 (59.52)	2,158 (53.56)	0.001
Current smoking	971 (20.89)	174 (28.06)	797 (19.78)	<0.001
Current drinking	747 (16.07)	115 (18.55)	632 (15.69)	0.071
Physical activity	3,902 (83.93)	519 (83.71)	3,383 (83.97)	0.871
Statin	377 (8.11)	57 (9.19)	320 (7.94)	0.335
β-Blocker	281 (6.04)	47 (7.58)	234 (5.81)	0.105
Hormones	21 (0.45)	4 (0.65)	17 (0.42)	0.653
ASCVD	728 (15.66)	117 (18.87)	611 (15.17)	0.018
Diabetes	1,640 (35.28)	277 (44.68)	1,363 (33.83)	<0.001
Hypertension	3,454 (74.30)	496 (80.00)	2,958 (73.42)	<0.001
Liver dysfunction	224 (4.82)	43 (6.94)	181 (4.49)	0.008
Homocysteine (μmol/L)	14.90 (12.60–18.00)	16.30 (13.70–19.78)	14.60 (12.50–17.70)	<0.001
SBP (mmHg)	141.98 (20.72)	142.77 (19.65)	141.85 (20.88)	0.298
DBP (mmHg)	80.45 (10.75)	80.63 (10.65)	80.44 (10.70)	0.685
HbAIc (%)	6.00 (5.80–6.50)	6.20 (5.80–6.80)	6.00 (5.80–6.40)	<0.001
FPG (mmol/L)	5.38 (4.94–6.24)	5.65 (5.07–6.96)	5.36 (4.93–6.14)	<0.001
TC (mg/dl)	192 (48)	171 (35)	195 (49)	<0.001
TG (mg/dl)	124 (91–172)	195 (141–274)	118 (88–159)	<0.001
LDL-C (mg/dl)	127 (37)	112 (32)	129 (37)	<0.001
ALT (U/L)	16 (12–21)	18 (13–24)	15 (12–21)	<0.001
AST (U/L)	20 (18–24)	20 (17–24)	20 (18–24)	0.049
UN (mg/dl)	15.78 (4.41)	15.78 (4.81)	15.78 (4.35)	0.977
UA (mg/dl)	5.63 (1.46)	6.17 (1.55)	5.54 (1.43)	<0.001
SCr (mg/dl)	0.87 (0.25)	0.93 (0.29)	0.86 (0.24)	<0.001
CCr (ml/min)	66.47 (18.76)	68.70 (18.12)	66.13 (18.84)	0.002
eGFR (C-MDRD) (ml/min/1.73 m^2^)				0.001
60<	126 (2.71)	25 (4.03)	101 (2.51)	
60–90	1,198 (25.77)	189 (30.48)	1,009 (25.04)	
≥90	3,273 (70.40)	400 (64.52)	2,873 (71.31)	
eGFR (CKD-EPI) (ml/min/1.73 m^2^)				<0.001
60 <	570 (12.26)	105 (16.94)	465 (11.54)	
60–90	3,087 (66.40)	401 (64.68)	2,686 (66.67)	
≥90	940 (20.22)	108 (17.42)	832 (20.65)	

*Data are mean (standard deviation), median (interquartile range), or frequency (percentage). Hormones refers to hormone replacement therapy for postmenopausal females as estrogen and/or progestin*.

*HDL-C, high-density lipoprotein cholesterol; BMI, body mass index; WC, waist circumference; ASCVD, atherosclerotic cardiovascular disease; SBP, systolic blood pressure; DBP, diastolic blood pressure; HbAIc, hemoglobin AIc; FPG, fasting plasma glucose; TC, total cholesterol; TG, triglycerides; LDL-C, low-density lipoprotein cholesterol; ALT, alanine aminotransferase; AST, aspartate aminotransferase; UN, urea nitrogen; UA, uric acid; SCr, serum creatinine; CCr, creatinine clearance; eGFR, estimated glomerular filtration rate; C-MDRD, Chinese Modification of Diet in Renal Disease; CKD-EPI, Chronic Kidney Disease Epidemiology Collaboration*.

### HDL-C and Renal Function

The preliminary exploration of HDL-C and renal function is presented in Table 2. HDL-C was positively correlated with UN, UN/SCr, and eGFR, while negatively correlated with UA, SCr, and CCr (*P* ≤ 0.010). Although there were significant correlations between HDL-C and renal function, all were relatively weak. Therefore, further analysis was performed.

**Table 2:** Pearson's correlation coefficients between HDL-C and renal function.

	**HDL-C**		
	***r***	**95% CI**	***P-*value**
UA	−0.261	−0.289 ~−0.236	<0.001
UN	0.038	0.010 ~ 0.069	0.010
SCr	−0.140	−0.167 ~−0.114	<0.001
UN/SCr	0.173	0.143 ~ 0.203	<0.001
CCr	−0.153	−0.181 ~−0.122	<0.001
eGFR, C-MDRD	0.047	0.017 ~ 0.080	0.002
eGFR, CKD-EPI	0.048	0.017 ~ 0.079	0.001

*UN, urea nitrogen; UA, uric acid; SCr, serum creatinine; BMI, body mass index; ASCVD, atherosclerotic cardiovascular disease; CCr, creatinine clearance; eGFR, estimated glomerular filtration rate; C-MDRD, Chinese Modification of diet in renal disease; CKD-EPI, chronic kidney disease epidemiology collaboration; CI, confidence interval*.

### eGFR and Low HDL-C

**Figure 2:**
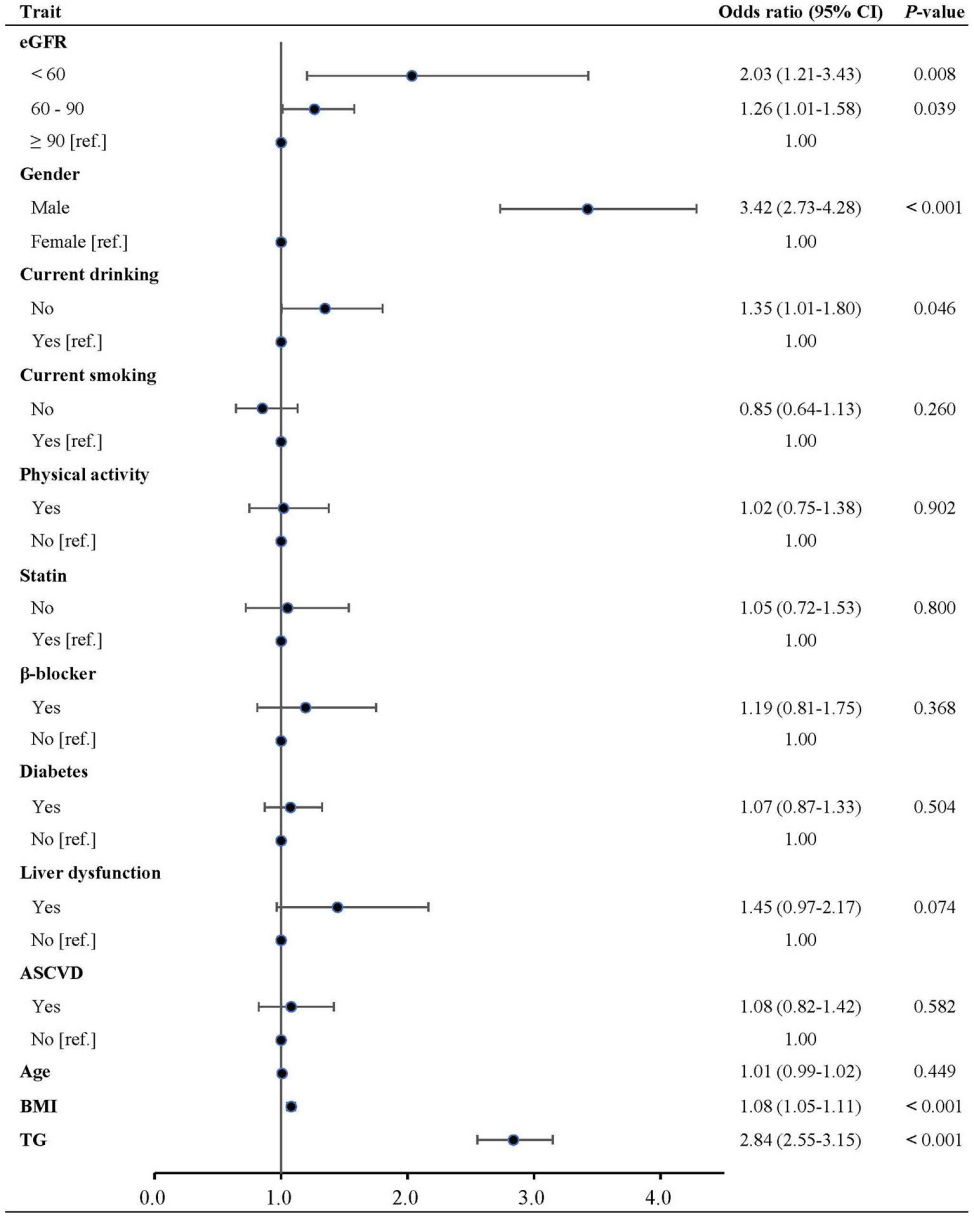
Multivariable-adjusted odds ratios of low HDL-C in participant subgroups. HDL-C, high-density lipoprotein cholesterol; eGFR, estimated glomerular filtration rate; BMI, body mass index; ASCVD, atherosclerotic cardiovascular disease; TG, triglycerides.

**Figure 3:**
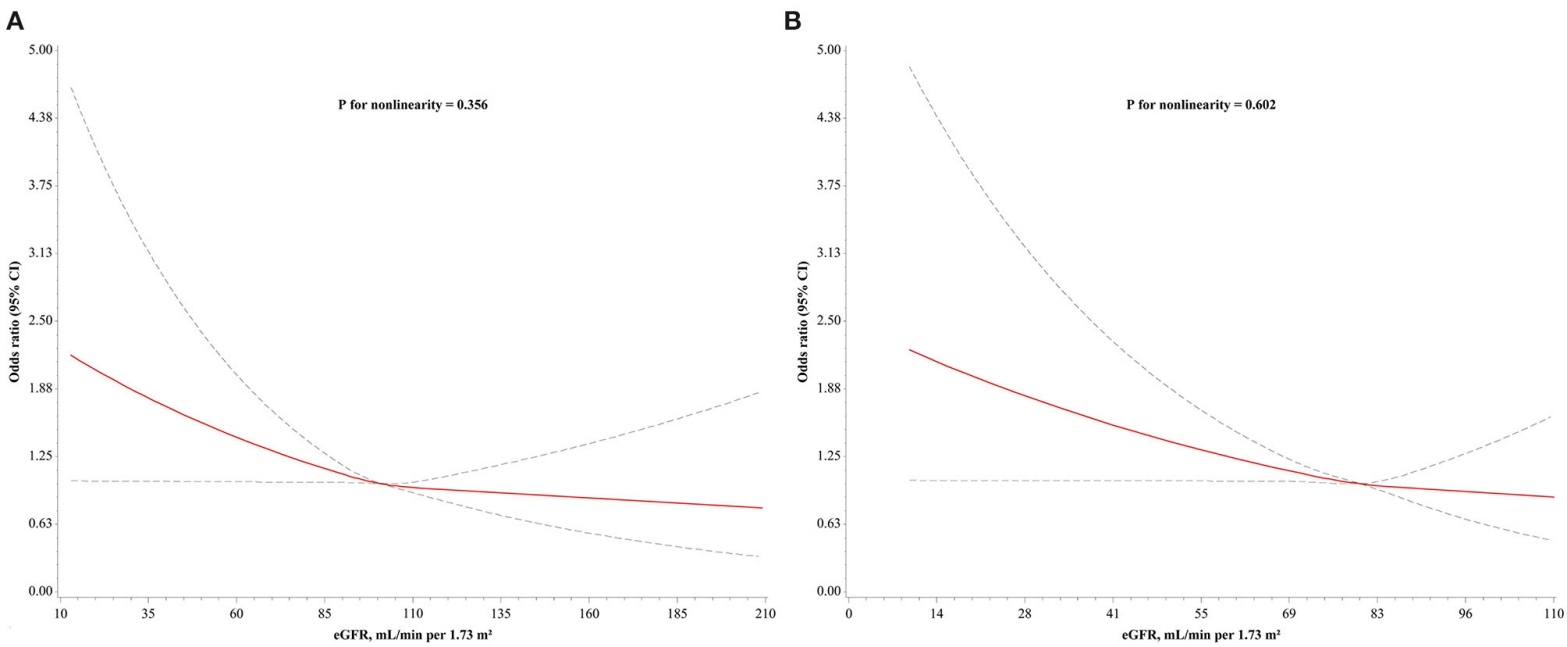
Adjusted dose-response relationship between low HDL-C and eGFR. **(A)** eGFR calculated using C-MDRD equation; **(B)** eGFR calculated using CKD-EPI equation. Odds ratios (95% CI) were obtained from restricted cubic splines with knots placed at 25, 50, and 75th percentiles of eGFR distribution. Reference points were median values of eGFR (**A**: 101 ml/min/1.73 m^2^; **B**: 80 ml/min/1.73 m^2^). Models were adjusted for age, gender, BMI, smoking, drinking, physical activity, β-blocker, statin, diabetes, liver dysfunction, ASCVD, and triglycerides. Solid line represents odds ratios and dotted lines represent 95% CI. HDL-C, high-density lipoprotein cholesterol; eGFR, estimated glomerular filtration rate; C-MDRD, Chinese Modification of Diet in Renal Disease; CKD-EPI, Chronic Kidney Disease Epidemiology Collaboration; BMI, body mass index; ASCVD, atherosclerotic cardiovascular disease; CI, confidence interval.

Decreased eGFR (C-MDRD) showed a significant independent association with low HDL-C. Compared with normal eGFR, the unadjusted odds ratios (OR) for lower and marginal eGFR were 1.78 (95% CI, 1.13–2.79) and 1.35 (95% CI, 1.12–1.62), respectively (Model I). The association was further strengthened, and the ORs were 1.81 (95% CI, 1.13–2.90) and 1.33 (95% CI, 1.10–1.62), respectively, after adjustment for age, gender, and BMI only (Model II). The association with low HDL-C was significantly higher in lower (OR, 2.03; 95% CI, 1.21–3.43) and marginal eGFR (OR, 1.26; 95% CI, 1.01–1.58) after further adjustment for lifestyles, medication and disease history, and triglycerides (Model III). eGFR, as a continuous variable, was significantly associated with low HDL-C (unadjusted OR (95% CI) per SD decrease: in Model I: 1.15 (1.05, 1.25), *P* = 0.002; multivariable-adjusted OR (95% CI): in Model II: 1.16 (1.06, 1.27), *P* = 0.001; in Model III: 1.17 (1.06, 1.30), *P* = 0.002) (Table 3 and Figure 2). To further evaluate whether the relationship between eGFR and low HDL-C was linear, a restricted cubic spline model was fitted, and no evidence of non-linearity was found (*P* for non-linearity, 0.356, Figure 3A).

**Table 3:** Odds ratios (95% CI) for eGFR, calculated by C-MDRD equation, in relation with low HDL-C.

**eGFR (ml/min/1.73 m^**2**^, median)**	**Model I**		**Model II**		**Model III**	
	**Odds ratio (95% CI)**	***P*-value**	**Odds ratio (95% CI)**	***P*-value**	**Odds ratio (95% CI)**	***P*-value**
Per SD decrease^*^	1.15 (1.05–1.25)	0.002	1.16 (1.06–1.27)	0.001	1.17 (1.06–1.30)	0.002
60< (53.16)	1.78 (1.13–2.79)	0.012	1.81 (1.13–2.90)	0.013	2.03 (1.21–3.43)	0.008
60–90 (80.75)	1.35 (1.12–1.62)	0.002	1.33 (1.10–1.62)	0.004	1.26 (1.01–1.58)	0.039
≥90 (108.49)	1.00 [ref.]		1.00 [ref.]		1.00 [ref.]	
Trend	*P* < 0.001		*P* < 0.001		*P* = 0.002	

*Model I: unadjusted. Model II: adjusted for age, gender, and BMI. Model III: as for Model II plus smoking, drinking, physical activity, β-blocker, statin, diabetes, liver dysfunction, ASCVD, and triglycerides*.

*SD, standard deviation; CI, confidence interval; BMI, body mass index; ASCVD, atherosclerotic cardiovascular disease; HDL-C, high-density lipoprotein cholesterol; eGFR, estimated glomerular filtration rate; C-MDRD, Chinese Modification of Diet in Renal Disease*.

* *One SD is equal to 22.6*.

### Subsidiary Analyses

To further confirm the findings, the CKD-EPI equation was used instead of the C-MDRD equation to calculate eGFR for verification (27). The results revealed that the association of low HDL-C with lower eGFR was significantly increased compared with normal eGFR in any of the models, and the ORs were increased with adjustment for lipid metabolism confounders (OR (95% CI): in Model I: 1.74 (1.30, 2.33), *P* < 0.001; in Model II: 2.05 (1.46, 2.88), *P* < 0.001; in Model III: 2.05 (1.44, 2.92), *P* < 0.001) (Table 4). However, the association of low HDL-C with marginal eGFR was not statistically significant compared with normal eGFR in any of the models (all *P* > 0.1, Table 4). Notably, the strength of association and non-linear relationship between eGFR, as a continuous variable, and low HDL-C remained generally consistent [fully adjusted OR (95% CI), 1.17 (1.06, 1.29), Table 4; *P* for non-linearity, 0.602, Figure 3B].

**Table 4:** Odds ratios (95% CI) for eGFR, calculated by CKD-EPI equation, in relation with low HDL-C.

**eGFR (ml/min/1.73 m^**2**^, median)**	**Model I**		**Model II**		**Model III**	
	**Odds ratio (95% CI)**	***P*-value**	**Odds ratio (95% CI)**	***P*-value**	**Odds ratio (95% CI)**	***P*-value**
Per SD decrease^*^	1.17 (1.08–1.27)	<0.001	1.21 (1.10–1.33)	<0.001	1.17 (1.06–1.29)	0.001
60< (51.97)	1.74 (1.30–2.33)	<0.001	2.05 (1.46–2.88)	<0.001	2.05 (1.44–2.92)	<0.001
60–90 (78.03)	1.15 (0.92–1.44)	0.226	1.19 (0.93–1.52)	0.159	1.23 (0.93–1.62)	0.141
≥90 (92.66)	1.00 [ref.]		1.00 [ref.]		1.00 [ref.]	
Trend	*P* < 0.001		*P* < 0.001		*P* < 0.001	

*Model I: unadjusted. Model II: adjusted for age, gender, and BMI. Model III: as for Model II plus smoking, drinking, physical activity, β-blocker, statin, diabetes, liver dysfunction, ASCVD, and triglycerides*.

*SD, standard deviation; CI, confidence interval; BMI, body mass index; ASCVD, atherosclerotic cardiovascular disease; HDL-C, high-density lipoprotein cholesterol; eGFR, estimated glomerular filtration rate; CKD-EPI, Chronic Kidney Disease Epidemiology Collaboration*.

* *One SD is equal to 14.4*.

## Discussion

This study found that eGFR was significantly associated with low HDL-C in community dwelling elderly, and this relationship was independent of identified HDL-C confounders, including age, gender, BMI, smoking, drinking, physical activity, β-blocker, statin, diabetes, liver dysfunction, ASCVD, and triglycerides. The findings also confirm some of the associations previously found in population studies: HDL-C levels were lower in males than females (29, 30), decreased with increasing BMI and triglycerides (31, 32), and were higher in individuals drinking alcohol (33, 34).

The decrease in eGFR is thought to lead to low HDL-C, which may be related to the significant changes in proteomics and lipidomics. Vaziri et al. found ApoA-I levels in ESRD patients were significantly decreased, due to both impaired synthesis and enhanced catabolism of ApoA-I in CKD patients. ApoA-I is an essential component of HDL particles, and its concentration decreases in CKD leading to a decrease in HDL-C (35). Meanwhile, the lack of hepatic triacylglycerol lipase in CKD patients leads to a decrease in cholesterol in HDL particles and an increase in triacylglycerol (36). On the other hand, oxidative stress and inflammatory reactions, uremic toxins, etc. in CKD patients cause denaturation, oxidation, and carbamylation of ApoA-I and other protein components of HDL particles, which affects the binding of HDL to cell surface cholesterol transporters, resulting in a decrease in HDL's ability to promote cholesterol efflux (37, 38). This in turn impairs the reverse cholesterol transport, and consequently promotes foam cell formation, accelerated atherosclerosis, endothelial dysfunction, oxidative stress, systemic inflammation, and glomerulosclerosis, which would exacerbate the occurrence and development of CVD or ESRD (12). Thus, it might be predicted that low HDL-C would be associated with decreased eGFR.

Conversely, previous studies have shown that low HDL-C can predict a deterioration in renal function (16–19). Low HDL-C could independently predict an increase in renal dysfunction in 840 patients with different kidney diseases, from the MDRD study (16), which was consistent with the result of the Atherosclerosis Risk in Communities (ARIC) cohort study (17). Bowe et al. used the U.S. Veterans Administration (VA) databases to conduct a retrospective cohort study of nearly 2 million male veterans and found that low HDL-C was significantly associated with the risk of incident kidney disease and its progression (18). Meanwhile, it has been reported that low HDL-C may be a surrogate marker of poor overall metabolic health (18). From our findings, it could be suggested to some extent that even in the general population, decreased eGFR may contribute to poor overall metabolic health and thus indirectly enhance CVD. Lees et al. incorporated eGFR into the traditional CVD risk prediction model and found that eGFR significantly enhanced the risk prediction ability of the models (39), which somewhat reflected the results of the present study. Additionally, data from UK Biobank showed that just examining total cholesterol and HDL-C could predict the impact of blood lipids on CVD risk (40). Consequently, in the general population, especially those with renal dysfunction, more attention should be paid to HDL-C levels, which may help predict the development of CVD as early as possible.

The association of low HDL-C with marginal eGFR presented different results in the analysis using C-MDRD and CKD-EPI equations, which may be attributed to differences in the original populations used to develop the equations. The cohort used to develop the MDRD equation was CKD patients, and the relationship between GFR and SCr concentrations is different among healthy and CKD individuals (41). Consequently, it is not unexpected to observe that the MDRD equation systematically underestimates GFR at high GFR levels (>60 ml/min/1.73 m^2^). This systematic underestimation at the population level leads to overestimations of CKD stage III prevalence (eGFR <60 ml/min/1.73 m^2^) in the general population. However, the population used to develop the CKD-EPI equation was mostly individuals with GFR > 60 ml/min/1.73 m^2^, which somewhat corrected the bias of the MDRD equation (27). Hence, the performance of the CKD-EPI equation was particularly improved for individuals with GFR >60 ml/min/1.73 m^2^, which was also seen in the European elderly population (42). However, it is noteworthy that the CKD-EPI equation provided only marginal improvement in precision compared with the MDRD equation (43, 44). Murata et al. compared the accuracy of the MDRD and CKD-EPI creatinine equations for estimating GFR in 5,238 patients and confirmed that the CKD-EPI creatinine equation underestimated GFR to a lesser extent than the MDRD equation in pre- and post-donation kidney donors. However, in patients with native CKD, renal transplant recipients and other organ recipients, the CKD-EPI equation did not perform better or was even slightly worse than the MDRD equation with a trend to overestimation (45). This overestimation in CKD patients could be the price of improved performance at higher GFR levels (44, 46). Furthermore, van den Brand et al. investigated 6,097 Caucasian participants and calculated eGFR using the MDRD and CKD-EPI equations and found that the CKD-EPI had higher estimates of GFR for subjects aged <70 years compared with the MDRD equation, but lower estimates for those aged 70 years and older (47). Hence, it might be considered that MDRD and CKD-EPI equations help to complement each other for validation in epidemiological studies.

This study had several strengths, including a large natural population of older participants, numerous confounders, medical record review to determine disease status and use of the China-specific eGFR definition. These all help in reducing bias.

Limitations of the present study should be considered. Firstly, given the nature of cross-sectional studies, it remains unclear whether the association between eGFR and low HDL-C changes over time, and therefore the causality between the two cannot be determined. Secondly, although confounders affecting HDL-C levels have been considered as much as possible, residual unmeasured confounders may remain. Thirdly, all participants were older adults in the Chinese community, making it difficult to generalize to different ethnic populations. Furthermore, over 70% of subjects in this sample had normal renal function according to the C-MDRD equation, which was markedly higher than the elderly population in the Caucasian community (48). Though previous studies have illustrated that the prevalence of CKD was lower in Asians than in Caucasians after adjusting for sex and age (49), considering that GFR of individuals decreases with age, the possibility that this may be an artifact of the C-MDRD equation could not be ruled out. Lastly, given the nature of epidemiological studies, multiple assessments of renal functional status were not achievable. Though poor eGFR may represent an episode of acute kidney injury, this is less likely in community populations. There are limitations to the estimation of GFR in older adults regardless of equations applied, but importantly, these equations are all frequently used in the clinical setting so that understanding the association between eGFR and lipids remains relevant. It is also reassuring that, despite these limitations, similar strength of association and dose-response relationships existed between renal dysfunction and low HDL-C, irrespective of methods used to define renal dysfunction.

## Conclusion

In this general elderly population, renal dysfunction was strongly associated with low HDL-C, independent of established HDL-C confounders. There was a dose-response relationship between low HDL-C and eGFR, with low HDL-C frequency increasing with decreasing eGFR. These findings raise the possibility that even slight changes in renal function may be associated with lipid levels in humans. Thus, it is speculated that there may be a reciprocal causality between renal dysfunction and lipid changes, which warrants further exploration in prospective studies.

## Data Availability Statement

The raw data supporting the conclusions of this article will be made available by the authors, without undue reservation.

## Ethics Statement

The studies involving human participants were reviewed and approved by Medical Ethics Committee of Tongji University Affiliated Shanghai East Hospital (2017-010). The patients/participants provided their written informed consent to participate in this study.

## Author Contributions

AY was responsible for conceptualization, formal analysis, and writing the manuscript. BT and YZ were responsible for revising the manuscript. LY was responsible for investigation and data collection. HF, ZL, YZ, and LZ were responsible for resources, supervision, and project administration. LZ was responsible for funding acquisition. All authors have read and approved the final version of the manuscript.

## Conflict of Interest

The authors declare that the research was conducted in the absence of any commercial or financial relationships that could be construed as a potential conflict of interest.
