# Supplementary material for: Association Between Renal Dysfunction and Low HDL Cholesterol Among the Elderly in China
Source: Front Cardiovasc Med. 2021 May 12;8:644208. doi: 10.3389/fcvm.2021.644208 (PMC8149893; doi:10.3389/fcvm.2021.644208)
Supplement: Supplementary file 1 [file Data_Sheet_1.docx]

**Sensitivity Analyses**

Sensitivity analyses were performed to (1) evaluate the relationship between eGFR and HDL-C as continuous variables using multiple linear regression models; (2) reassess renal function using the full age spectrum (FAS) equation, which has proved to be more appropriate for older adults(1); (3) change the threshold for low HDL-C to <40 mg/dL for males and <50 mg/dL for females(2); and (4) delineate renal function using age-dependent eGFR cutoffs(3).

**Results**

HDL-C was significantly associated with eGFR (eGFR: C-MDRD: β = 0.034, 95% CI: 0.016 - 0.053, *P* < 0.001, Appendix Table 1; CKD-EPI: β = 0.047, 95% CI: 0.016 - 0.078, *P* = 0.003, Appendix Table 2). HDL-C increased by 0.034 mg/dL (C-MDRD equation) and 0.047 mg/dL (CKD-EPI equation) per unit change in eGFR, respectively.

Appendix Table 1. Multiple linear regression analysis of HDL-C and eGFR using the C-MDRD equation.

|  | β | 95% CI | SE | *P*-value | Collinearity | |
| --- | --- | --- | --- | --- | --- | --- |
|  |  |  |  |  | Tolerance | VIF |
| Constant | 72.937 | 66.119 ~ 79.754 | 3.477 | < 0.001 |  |  |
| Age, years | 0.023 | -0.042 ~ 0.087 | 0.033 | 0.496 | 0.878 | 1.138 |
| Gender | 8.065 | 7.122 ~ 9.008 | 0.481 | < 0.001 | 0.698 | 1.432 |
| BMI, kg/m^2^ | -0.943 | -1.060 ~ -0.825 | 0.060 | < 0.001 | 0.937 | 1.068 |
| Current smoking | -1.009 | -2.270 ~ 0.252 | 0.643 | 0.117 | 0.623 | 1.605 |
| Current drinking | 2.325 | 0.988 ~ 3.661 | 0.682 | 0.001 | 0.706 | 1.417 |
| Physical activity | -0.134 | -1.286 ~ 1.018 | 0.588 | 0.819 | 0.996 | 1.004 |
| β-blocker | -1.334 | -2.984 ~ 0.315 | 0.841 | 0.113 | 0.948 | 1.055 |
| Statin | 0.205 | -1.289 ~ 1.698 | 0.762 | 0.788 | 0.893 | 1.120 |
| Diabetes | -1.880 | -2.729 ~ -1.030 | 0.433 | < 0.001 | 0.932 | 1.073 |
| Liver dysfunction | 2.637 | -0.382 ~ 5.656 | 1.540 | 0.087 | 0.996 | 1.004 |
| ASCVD | -1.073 | -2.185 ~ 0.039 | 0.567 | 0.059 | 0.917 | 1.091 |
| Triglycerides, mg/dL | -0.075 | -0.080 ~ -0.071 | 0.002 | < 0.001 | 0.946 | 1.057 |
| eGFR, mL/min/1.73 m^2^ | 0.034 | 0.016 ~ 0.053 | 0.009 | < 0.001 | 0.903 | 1.107 |

BMI, body mass index; ASCVD, atherosclerotic cardiovascular disease; eGFR, estimated glomerular filtration rate; CI, confidence interval; SE, standard error; VIF, variance inflation factor; C-MDRD, Chinese Modification of Diet in Renal Disease.

Appendix Table 2. Multiple linear regression analysis of HDL-C and eGFR using the CKD-EPI equation.

|  | β | 95% CI | SE | *P*-value | Collinearity | |
| --- | --- | --- | --- | --- | --- | --- |
|  |  |  |  |  | Tolerance | VIF |
| Constant | 72.015 | 64.414 ~ 79.615 | 3.877 | < 0.001 |  |  |
| Age, years | 0.035 | -0.034 ~ 0.105 | 0.036 | 0.320 | 0.759 | 1.317 |
| Gender | 7.958 | 7.014 ~ 8.901 | 0.481 | < 0.001 | 0.699 | 1.432 |
| BMI, kg/m^2^ | -0.942 | -1.060 ~ -0.825 | 0.060 | < 0.001 | 0.936 | 1.068 |
| Current smoking | -1.039 | -2.300 ~ 0.222 | 0.643 | 0.106 | 0.623 | 1.605 |
| Current drinking | 2.333 | 0.996 ~ 3.670 | 0.682 | 0.001 | 0.706 | 1.417 |
| Physical activity | -0.135 | -1.288 ~ 1.018 | 0.588 | 0.818 | 0.996 | 1.004 |
| β-blocker | -1.336 | -2.987 ~ 0.315 | 0.842 | 0.113 | 0.948 | 1.055 |
| Statin | 0.192 | -1.302 ~ 1.687 | 0.762 | 0.801 | 0.893 | 1.120 |
| Diabetes | -1.822 | -2.671 ~ -0.973 | 0.433 | < 0.001 | 0.935 | 1.070 |
| Liver dysfunction | 2.647 | -0.374 ~ 5.667 | 1.541 | 0.086 | 0.996 | 1.004 |
| ASCVD | -1.070 | -2.182 ~ 0.043 | 0.568 | 0.060 | 0.917 | 1.091 |
| Triglycerides, mg/dL | -0.076 | -0.080 ~ -0.071 | 0.002 | < 0.001 | 0.946 | 1.057 |
| eGFR, mL/min/1.73 m^2^ | 0.047 | 0.016 ~ 0.078 | 0.016 | 0.003 | 0.780 | 1.282 |

BMI, body mass index; ASCVD, atherosclerotic cardiovascular disease; eGFR, estimated glomerular filtration rate; CI, confidence interval; SE, standard error; VIF, variance inflation factor; CKD-EPI, Chronic Kidney Disease Epidemiology Collaboration.

For the FAS equation, the fully adjusted odds ratios (ORs) for lower and marginal eGFR were 1.98 (95% CI, 1.24-3.15) and 1.57 (95% CI, 1.01-2.44), respectively. Interestingly, the OR for per SD decrease in eGFR was 1.17 regardless of the equation (OR, 1.17; 95% CI, 1.06-1.30; Appendix Table 3). Additionally, the non-linearity between low HDL-C and eGFR remained essentially unchanged (*P* for non-linearity, 0.489, Appendix Figure 1)

Appendix Table 3. Multiple logistic regression analysis of low HDL-C and eGFR using the FAS equation.

| eGFR (mL/min/1.73 m^2^, median) | Model I | | Model II | | Model III | |
| --- | --- | --- | --- | --- | --- | --- |
|  | OR (95% CI) | *P*-value | OR (95% CI) | *P*-value | OR (95% CI) | *P*-value |
| Per SD decrease* | 1.13 (1.04-1.23) | 0.005 | 1.21 (1.09-1.34) | < 0.001 | 1.17 (1.06-1.30) | 0.002 |
| 60< (52.50) | 1.56 (1.06-2.30) | 0.026 | 1.92 (1.26-2.94) | 0.003 | 1.98 (1.24-3.15) | 0.004 |
| 60 – 90 (72.22) | 1.32 (0.91-1.91) | 0.140 | 1.42 (0.97-2.08) | 0.071 | 1.57 (1.01-2.44) | 0.047 |
| ≥90 (95.85) | 1.00 [ref.] |  | 1.00 [ref.] |  | 1.00 [ref.] |  |
| Trend | *P =* 0.015 | | *P =* 0.001 | | *P =* 0.002 | |

Model I: unadjusted. Model II: adjusted for age, gender, and BMI. Model III: as for Model II plus smoking, drinking, physical activity, β-blocker, statin, diabetes, liver dysfunction, ASCVD, and triglycerides.

OR, odds ratio; SD, standard deviation; CI, confidence interval; BMI, body mass index; ASCVD, atherosclerotic cardiovascular disease; HDL-C, high density lipoprotein cholesterol; eGFR, estimated glomerular filtration rate; FAS, full age spectrum.

*One SD is equal to 14.9.


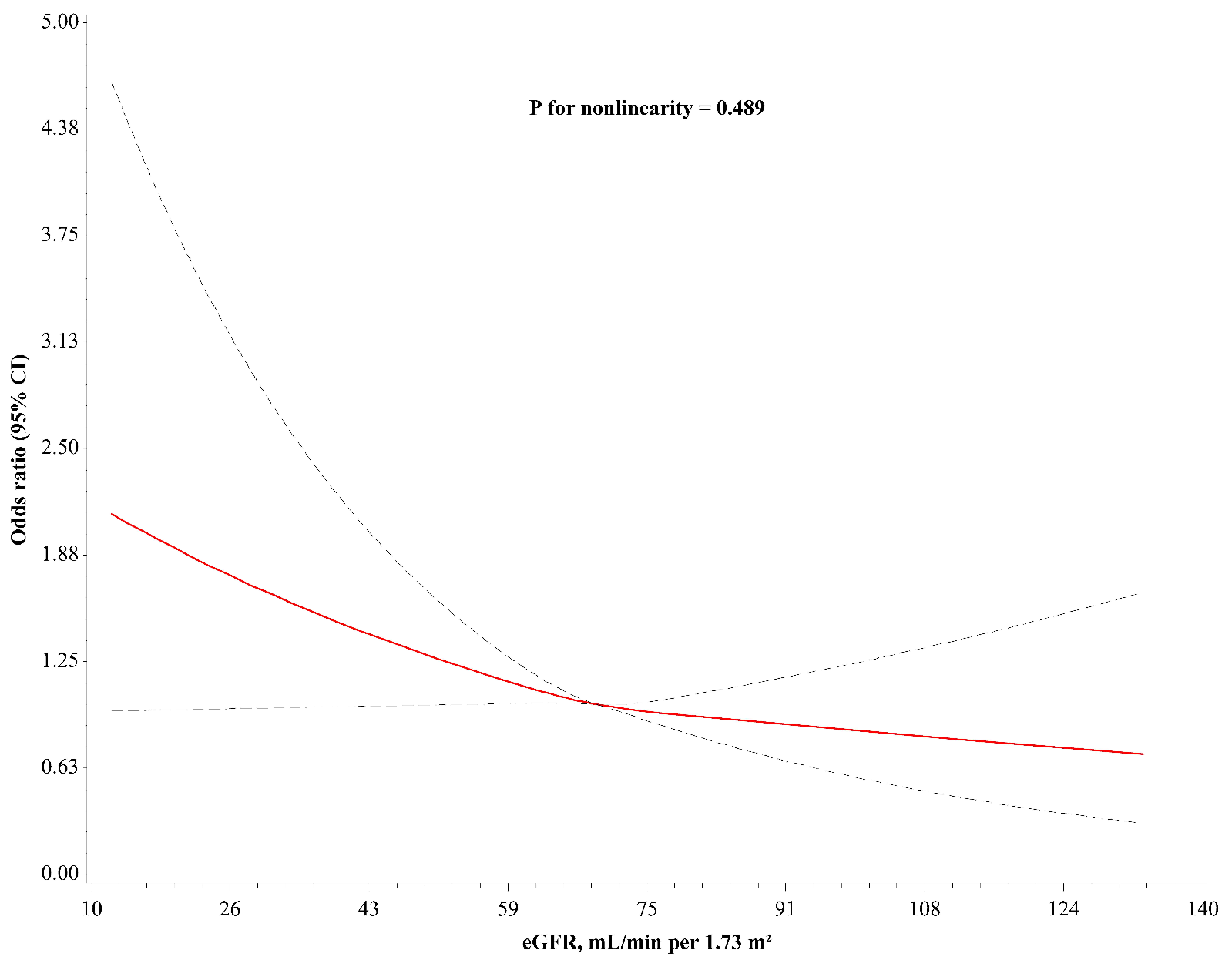
Appendix Figure 1. Adjusted dose-response relationship between low HDL-C and eGFR, calculated by FAS equation. Odds ratios (95% CI) were obtained from restricted cubic splines with knots placed at 25th, 50th, and 75th percentiles of eGFR distribution. Reference points were median values of eGFR (69 mL/min/1.73 m^2^). Model was adjusted for age, gender, BMI, smoking, drinking, physical activity, β-blocker, statin, diabetes, liver dysfunction, ASCVD, and triglycerides. Solid line represents odds ratios and dotted lines represent 95% CI. eGFR, estimated glomerular filtration rate; HDL-C, high density lipoprotein cholesterol; FAS, full age spectrum; BMI, body mass index; ASCVD, atherosclerotic cardiovascular disease; CI, confidence interval.

When low HDL-C thresholds were altered, the ORs for renal dysfunction compared with normal renal function was 1.87 (95% CI, 1.20-2.91) and 1.50 (95% CI, 1.12-2.01), respectively, after adjusting for demographics, lifestyles, medication and disease history, and triglycerides (Appendix Table 4).

Appendix Table 4. Multiple logistic regression analyses of eGFR and low HDL-C (<40 mg/dL for males; <50 mg/dL for females).

| eGFR, mL/min/1.73 m^2^ | Model I | | Model II | | Model III | |
| --- | --- | --- | --- | --- | --- | --- |
|  | OR (95% CI) | *P*-value | OR (95% CI) | *P*-value | OR (95% CI) | *P*-value |
| C-MDRD |  |  |  |  |  |  |
| Per SD decrease† | 1.09 (1.02-1.15) | 0.006 | 1.15 (1.07-1.23) | < 0.001 | 1.10 (1.02-1.19) | 0.018 |
| 60< | 1.60 (1.12 - 2.29) | 0.009 | 1.83 (1.24-2.70) | 0.002 | 1.87 (1.20-2.91) | 0.006 |
| 60 - 90 | 1.11 (0.97-1.27) | 0.121 | 1.22 (1.05-1.42) | 0.008 | 1.13 (0.95-1.34) | 0.176 |
| ≥90 | 1.00 [ref.] |  | 1.00 [ref.] |  | 1.00 [ref.] |  |
| CKD-EPI |  |  |  |  |  |  |
| Per SD decrease‡ | 1.10 (1.03-1.16) | 0.002 | 1.17 (1.09-1.26) | < 0.001 | 1.11 (1.03-1.21) | 0.010 |
| 60< | 1.32 (1.07-1.63) | 0.011 | 1.65 (1.28-2.13) | < 0.001 | 1.50 (1.12-2.01) | 0.007 |
| 60 - 90 | 1.16 (1.00-1.35) | 0.048 | 1.23 (1.04-1.45) | 0.014 | 1.07 (0.88-1.29) | 0.514 |
| ≥90 | 1.00 [ref.] |  | 1.00 [ref.] |  | 1.00 [ref.] |  |

Model I: unadjusted. Model II: adjusted for age, gender, and BMI. Model III: as for Model II plus smoking, drinking, physical activity, β-blocker, statin, diabetes, liver dysfunction, ASCVD, and triglycerides.

OR, odds ratio; SD, standard deviation; CI, confidence interval; BMI, body mass index; ASCVD, atherosclerotic cardiovascular disease; HDL-C, high density lipoprotein cholesterol; eGFR, estimated glomerular filtration rate; C-MDRD, Chinese Modification of Diet in Renal Disease; CKD-EPI, Chronic Kidney Disease Epidemiology Collaboration.

†One SD is equal to 22.6.

‡One SD is equal to 14.4.

Age-adapted definition of CKD was used for sensitivity analysis, considering the normal decline of renal function in the elderly. The results revealed that for the C-MDRD and CKD-EPI equations, no significant association between eGFR < 45 mL/min/1.73m^2^ and low HDL-C was observed. However, for the FAS equation, for eGFR < 45 mL/min/1.73m^2^ was significantly associated with low HDL-C. Moreover, for eGFR in the range of 45-60 mL/min/1.73m^2^, there were significant associations of moderate strength with low HDL-C (ORs (95% CI): C-MDRD: 2.25 (1.27-3.98); CKD-EPI: 1.79 (1.31-2.45); FAS: 1.42 (1.07-1.89); Appendix Table 5).

Appendix Table 5. Multiple logistic regression analyses of low HDL-C and eGFR (age-dependent cutoffs).

| eGFR, mL/min/1.73 m^2^ | Model I | | Model II | | Model III | |
| --- | --- | --- | --- | --- | --- | --- |
|  | OR (95% CI) | *P*-value | OR (95% CI) | *P*-value | OR (95% CI) | *P*-value |
| C-MDRD |  |  |  |  |  |  |
| 45< | 1.09 (0.38-3.15) | 0.870 | 0.86 (0.25-2.91) | 0.808 | 0.98 (0.27-3.57) | 0.978 |
| 45 - 60 | 1.82 (1.10-3.01) | 0.019 | 1.94 (1.16-3.26) | 0.011 | 2.25 (1.27-3.98) | 0.006 |
| 60 - 75 | 1.51 (1.13-2.01) | 0.005 | 1.59 (1.18-2.14) | 0.002 | 1.50 (1.06-2.13) | 0.022 |
| ≥75 | 1.00 [ref.] |  | 1.00 [ref.] |  | 1.00 [ref.] |  |
| CKD-EPI |  |  |  |  |  |  |
| 45< | 1.39 (0.87-2.23) | 0.167 | 1.51 (0.91-2.51) | 0.114 | 1.49 (0.86-2.56) | 0.152 |
| 45 - 60 | 1.67 (1.28-2.17) | < 0.001 | 1.89 (1.40-2.54) | < 0.001 | 1.79 (1.31-2.45) | < 0.001 |
| 60 - 75 | 1.09 (0.89-1.33) | 0.409 | 1.06 (0.86-1.31) | 0.598 | 0.95 (0.75-1.21) | 0.693 |
| ≥75 | 1.00 [ref.] |  | 1.00 [ref.] |  | 1.00 [ref.] |  |
| FAS |  |  |  |  |  |  |
| 45< | 1.53 (1.06-2.20) | 0.023 | 1.92 (1.25-2.97) | 0.003 | 1.74 (1.12-2.68) | 0.013 |
| 45 - 60 | 1.35 (1.06-1.71) | 0.016 | 1.61 (1.22-2.12) | 0.001 | 1.42 (1.07-1.89) | 0.014 |
| 60 - 75 | 1.27 (1.04-1.56) | 0.022 | 1.32 (1.06-1.63) | 0.012 | 1.24 (0.97-1.58) | 0.083 |
| ≥75 | 1.00 [ref.] |  | 1.00 [ref.] |  | 1.00 [ref.] |  |

Model I: unadjusted. Model II: adjusted for age, gender, and BMI. Model III: as for Model II plus smoking, drinking, physical activity, β-blocker, statin, diabetes, liver dysfunction, ASCVD, and triglycerides.

OR, odds ratio; SD, standard deviation; CI, confidence interval; BMI, body mass index; ASCVD, atherosclerotic cardiovascular disease; HDL-C, high density lipoprotein cholesterol; eGFR, estimated glomerular filtration rate; C-MDRD, Chinese Modification of Diet in Renal Disease; CKD-EPI, Chronic Kidney Disease Epidemiology Collaboration; FAS, full age spectrum.

**REFERENCES**1. Pottel H, Hoste L, Dubourg L, Ebert N, Schaeffner E, Eriksen BO, et al. An estimated glomerular filtration rate equation for the full age spectrum. *Nephrol Dial Transplant* (2016) 31(5):798-806. Epub 2016/03/05. doi: 10.1093/ndt/gfv454. PubMed PMID: 26932693; PubMed Central PMCID: PMCPMC4848755.

2. Stone NJ, Robinson JG, Lichtenstein AH, Bairey Merz CN, Blum CB, Eckel RH, et al. 2013 ACC/AHA guideline on the treatment of blood cholesterol to reduce atherosclerotic cardiovascular risk in adults: a report of the American College of Cardiology/American Heart Association Task Force on Practice Guidelines. *J Am Coll Cardiol* (2014) 63(25 Pt B):2889-934. Epub 2013/11/19. doi: 10.1016/j.jacc.2013.11.002. PubMed PMID: 24239923.

3. Delanaye P, Jager KJ, Bökenkamp A, Christensson A, Dubourg L, Eriksen BO, et al. CKD: A Call for an Age-Adapted Definition. *J Am Soc Nephrol* (2019) 30(10):1785-805. Epub 2019/09/12. doi: 10.1681/asn.2019030238. PubMed PMID: 31506289; PubMed Central PMCID: PMCPMC6779354.
